# Adhesion GPCRs in Kidney: New Insights into Development, Physiology and Disease

**DOI:** 10.3390/cells15181669

**Published:** 2026-09-15

**Authors:** Aruzhan Myrzatayeva, Abhishek K. Singh, Felix B. Engel

**Affiliations:** Experimental Renal and Cardiovascular Research, Department of Nephropathology, Institute of Pathology and Department of Cardiology, Friedrich-Alexander-Universität Erlangen-Nürnberg (FAU), 91054 Erlangen, Germany; aruzhan.myrzatayeva@uk-erlangen.de (A.M.); abhishek.singh@uk-erlangen.de (A.K.S.)

**Keywords:** adhesion G protein-coupled receptor, kidney, kidney disease, kidney development, Gα, signaling pathways

## Abstract

**Highlights:**

**What are the main findings?**

- Adhesion G protein-coupled receptors (aGPCRs) are potential therapeutic targets in kidney disease.
- Analysis of known non-renal functions of aGPCRs in context of their renal spatio-temporal expression allows prediction of potential renal functions.

**What is the implication of the main finding?**

- New tools are required to determine the spatio-temporal and cellular expression of aGPCRs.
- Studies modulating aGPCR function in renal disease models are needed to determine the therapeutic potential of aGPCRs.

**Abstract:**

Recently, adhesion G-protein-coupled receptors (aGPCRs) gained recognition for their essential roles in embryonic development. However, for the majority of them, their exact functions and underlying signaling mechanisms remain poorly understood. Here, we summarize the existing literature on aGPCRs in the context of kidney development, physiology, and disease. We compile available aGPCR expression profiles, discuss their validation statuses, and describe the knockout phenotypes. For instance, *Celsr1*/*Adgrc1* is expressed early in metanephric development, and its deletion results in aberrant oriented cell division and simplified ureteric trees. Furthermore, GPR116/ADGRF5 is important for the integrity of the glomerular filtration barrier and regulation of renal acid secretion. Several aGPCRs are also implicated in kidney diseases, e.g., deletion of *Gpr56*/*Adgrg1* ameliorates diabetic kidney disease. On the other hand, upregulation of GPR97/ADGRG3 exacerbates acute kidney injury and hypertensive nephropathy. Notably, for around two-thirds of the aGPCRs expressed in the kidney, no function has been proposed. For such aGPCRs, we draw hypotheses for potential functions based on their role in other tissues. Our goal is to shed light on how aGPCRs potentially contribute to the complex and highly regulated orchestration of cellular and molecular mechanisms underlying disease and development of a highly specialized organ such as the kidney.

## 1. Introduction

The kidney is essential for systemic homeostasis, continuously filtering the blood to regulate fluid and electrolyte balance, acid–base status, and the excretion of metabolic waste. In addition, it also acts as an endocrine organ that produces erythropoietin, renin, and the active form of vitamin D. These diverse functions rest on a highly ordered tissue architecture and on tightly regulated communication among specialized cell types, rendering the kidney sensitive to disturbances in cell–cell and cell–matrix interactions, epithelial polarity, and mechanotransduction.

The architecture of the kidney is established during development, when the metanephric kidney arises through reciprocal inductive signaling between the ureteric bud (UB), an epithelial outgrowth of the nephric duct, and the surrounding metanephric mesenchyme [1]. Beginning around embryonic day (E) 10.5 in the mouse (~week 5 in humans), “glial cell line-derived neurotrophic factor” (GDNF) secreted by the mesenchyme signals through the tyrosine kinase receptor “rearranged during transfection” (RET) at the UB tips to drive iterative branching morphogenesis—reinforced by “wingless-related integration site 11” (Wnt11) and “fibroblast growth factor 7” (FGF7) signaling—resulting in the collecting duct (CD) tree [1,2]. In parallel, the cap mesenchyme forms a self-renewing nephron progenitor pool that, in response to UB-derived Wnt9b and Wnt4, undergoes a mesenchymal-to-epithelial transition to build the nephron, with distinct components—Bowman’s capsule, proximal tubule (PT), loop of Henle (LOH), and distal tubule (DT)—patterned by the coordinated action of Wnt/β-catenin, FGF, and Notch signaling [1]. Tubule elongation and the control of tubular diameter depend on planar cell polarity (PCP) signaling, which orients cell division and drives convergent-extension movements along the tubule axis [3,4]. Notably, because the core PCP receptor Frizzled couples to heterotrimeric G proteins and the Gα_i_–LGN (leucine–glycine–asparagine)–NuMA (nuclear and mitotic apparatus) module positions the mitotic spindle, PCP-directed morphogenesis is mechanistically linked to G protein signaling and oriented cell division [5,6].

The mature human kidney contains on average around one million nephrons [7,8]. These functional units of the mature kidney contain structurally and molecularly distinct segments that together accomplish filtration, reabsorption, and secretion [9]. Filtration occurs at the renal corpuscle, where the glomerular filtration barrier (GFB)—a fenestrated endothelium, the glomerular basement membrane (GBM), and interdigitating podocyte foot processes bridged by slit diaphragms—generates a plasma ultrafiltrate while restricting passage by molecular size and charge; breakdown of this barrier permits protein leakage and manifests as proteinuria, a hallmark of glomerular disease [10,11]. The filtrate then enters the PT, whose dense brush border and high mitochondrial content drive bulk, largely iso-osmotic reabsorption of most filtered sodium and water together with virtually all glucose, amino acids, and bicarbonate—an energetic demand that also renders this segment especially vulnerable to ischemic and toxic injury. In the LOH, the water-permeable thin descending limb and the water-impermeable thick ascending limb, which actively reabsorbs NaCl via the renal Na^+^-K^+^-2Cl^−^ cotransporter (NKCC2), function as a countercurrent multiplier that establishes the medullary osmotic gradient required to concentrate urine. The distal convoluted tubule (DCT) subsequently fine-tunes sodium balance through the thiazide-sensitive sodium-chloride cotransporter (NCC) and governs calcium and magnesium handling, while the connecting tubule (CNT) and collecting ducts (CDs) carry out the final, hormonally regulated adjustment of sodium and potassium (via the epithelial sodium channel (ENaC) and principal cells), water reabsorption (via vasopressin-controlled aquaporin-2), and acid–base balance (via intercalated cells, IC), thereby setting the ultimate volume, osmolarity, and pH of the urine [9].

The epithelial and endothelial cells that make up the nephron segments are not passive conduits: they are continuously subjected to mechanical stimuli—fluid shear stress generated by glomerular filtration and tubular flow, hydrostatic pressure, and circumferential wall tension—which they convert into intracellular signals through mechanotransduction [12]. These forces are detected by a range of sensors, including the primary cilium acting as a flow-sensing antenna, stretch- and flow-activated ion channels, and integrin-based focal adhesions, and are relayed through calcium, purinergic, and nitric oxide signaling to modulate epithelial cell polarity, proliferation, survival, differentiation, and transepithelial transport [13,14,15,16,17]. Mechanosensation and adhesion-dependent signaling have accordingly emerged as central principles in kidney biology—operating in normal transport physiology and becoming dysregulated when renal hemodynamics are disturbed in disease.

The clinical stakes of advancing kidney biology are both high and rising. Chronic kidney disease (CKD) already affects more than one in ten individuals worldwide, and its prevalence is rising steeply, driven by increasing rates of type 2 diabetes and hypertension as well as population aging [18]. It now ranks among the fastest-growing causes of death and is projected to become the fifth leading cause of death globally by 2040 [19,20]. Acute kidney injury (AKI) frequently initiates or accelerates this progression, and—although recent agents such as SGLT2 inhibitors have improved outcomes—therapies that directly target the diverse molecular drivers of kidney injury remain limited [21,22]. Closing this gap will require the identification of novel, mechanism-based targets, making the systematic characterization of understudied yet druggable receptor families in the kidney a clear priority.

## 2. The Role of GPCRs in Kidney Development and Function

G protein-coupled receptors (GPCRs) are the largest superfamily of cell-surface membrane proteins in humans, encoded by ~800–1000 genes and characterized by a conserved seven-transmembrane (7TM) α-helical structure. They detect diverse extracellular cues such as photons, ions, neurotransmitters, hormones, lipids, peptides, as well as proteins, and transduce them into intracellular signals via heterotrimeric G proteins and other effectors [23]. GPCRs also play major roles in kidney development and function. In development, they are required for UB branching, nephron formation, and tubular morphogenesis, as recently reviewed [24,25,26]. In the adult kidney, they regulate water, electrolyte, and/or acid–base homeostasis (e.g., V2R [27,28], GPR39 [29,30], P2Y2R [31], CaSR [32], ADGRF5 [33]). Poll and co-workers have provided a detailed landscape of GPCR expression along the mouse nephron, identifying members of several GPCR classes including adrenergic receptors, lysophosphatidic acid receptors, olfactory receptors, and adhesion GPCRs (aGPCRs) [34].

**Figure 1:**
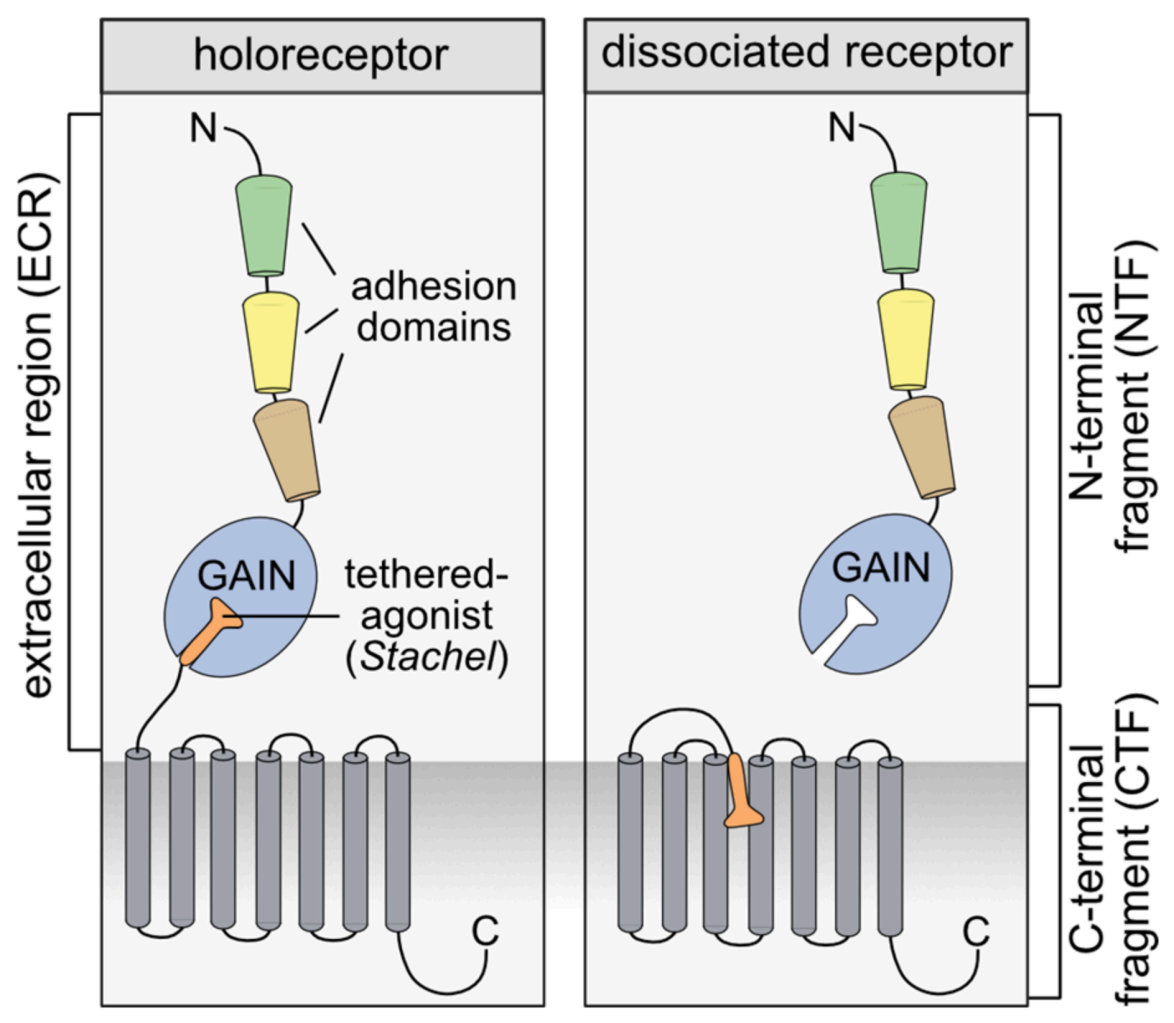
Schematic representation of the aGPCR holoreceptor (**left**) and its disassociated form (**right**). An aGPCR exhibits a long extracellular region (ECR) containing multiple adhesion domains and the conserved GPCR-autoproteolysis-inducing (GAIN) domain. The GAIN domain undergoes autoproteolysis during protein maturation, thereby generating an N-terminal and a C-terminal fragment (NTF and CTF). NTF and CTF remain non-covalently associated at the cell surface. The N-terminus of the CTF contains a tethered peptide agonist, called *Stachel*. The dissociation of the NTF from the CTF, via ligand binding or force, exposes the *Stachel* peptide, representing one of the activation mechanisms of aGPCRs.

Notably, a substantial proportion of aGPCRs is expressed in the kidney—during nephrogenesis, in the mature nephron, and in diseased tissue—yet for the great majority, their renal function remains essentially unexplored, and detailed mechanistic information exists for only a handful [35,36]. The aGPCRs are the second largest GPCR class, with 33 members in humans distributed across nine subfamilies. Their features make them compelling candidates for controlling kidney development and function. aGPCRs have an unusual architecture allowing activation by multiple mechanisms, including exogenous ligands, mechanical stimuli, and cell–cell interactions via other membrane proteins [37,38,39,40,41,42,43]. Each receptor carries an extended N-terminal ectodomain composed of varied adhesion modules, followed by a GPCR autoproteolysis-inducing (GAIN) domain and the 7TM core; autoproteolytic cleavage at the GPCR proteolysis site (GPS) within the GAIN domain generates an N-terminal fragment (NTF) and a C-terminal fragment (CTF) that typically remain non-covalently associated at the cell surface (Figure 1) [44].

NTF and CTF can have distinct independent functions [45,46,47]. Canonical activation depends on a cryptic tethered-agonist sequence—termed the “*Stachel*”—located at the newly formed N-terminus of the CTF: conformational change, dissociation of the NTF, or mechanical force can unmask this sequence, allowing it to engage the 7TM bundle and initiate signaling [48,49,50]. This force-gated, tethered-agonist mechanism is precisely what enables aGPCRs to convert mechanical and adhesive inputs into intracellular responses [42]. Once activated, aGPCRs couple to multiple heterotrimeric G proteins—Gα_s_, Gα_i/o_, Gα_q/11_, and G_α12/13_—to modulate 3′,5′-cyclic adenosine monophosphate (cAMP), calcium, and ras homolog family member A (RhoA)-dependent cytoskeletal remodeling, and several members additionally engage G protein-independent effectors, giving the family a broad and context-dependent signaling repertoire [35,44,51]. To unify the nomenclature of aGPCRs, the Adhesion GPCR Consortium together with the International Union of Basic and Clinical Pharmacology Committee on Receptor Nomenclature and Drug Classification (NC-IUPHAR) renamed aGPCRs with “ADGR” as a common denominator followed by a letter and a number denoting the subfamily and subtype, respectively [44].

Notably, aGPCRs hold considerable therapeutic promise. GPCRs as a whole are the most successfully druggable superfamily of proteins, accounting for roughly one-third of all approved medicines, so a large and disease-associated GPCR class represents an appealing reservoir of potential drug targets [52,53,54]. Furthermore, several preclinical and clinical studies underline the therapeutic potential of aGPCRs. For instance, a clinical study using aerosolized ADGRL3 antibody in pediatric asthma patients reported significant improvements in symptoms and inflammatory markers [55]. In addition, two clinical trials [NCT05463640, NCT05748197] utilize increased expression of the aGPCR EMR2/ADGRE2 for a CAR-T cell therapy to target and eliminate leukemic stem cells in acute myeloid leukemia patients [56,57]. In a preclinical study, structure-enabled in silico screening identified a small-molecule agonist of GPR133/ADGRD1, AP503, that enhances muscle strength but does not exhibit side effects mediated by the androgen 5α-dihydro-testosterone (5α-DHT), which acts in part through ADGRD1 [58]. 5α-DHT is used for androgen replacement therapy [59], but it has considerable adverse side effects, including an increased risk of prostate cancer [58]. Finally, targeting GPR110/ADGRF1 via its agonists synaptamide and dimethyl-synaptamide improved visual deficit in mouse models of optic nerve injury and mild traumatic brain injury [60,61].

aGPCRs are numerous, broadly or segment-specifically expressed across the developing and diseased kidney, and pharmacologically tractable, yet the function of most renal aGPCRs remains undefined, even as the burden of kidney disease continues to climb and disease-modifying therapies remain scarce. Bridging this gap could yield both fundamental insight into renal signal integration and new avenues for therapeutic intervention. Here, we review the current knowledge of aGPCRs in kidney development, physiology, and disease, with emphasis on receptor expression, signaling mechanisms, experimental model systems, and translational potential.

## 3. aGPCRs in Renal Development, Physiology, and Disease

**Figure 2:**
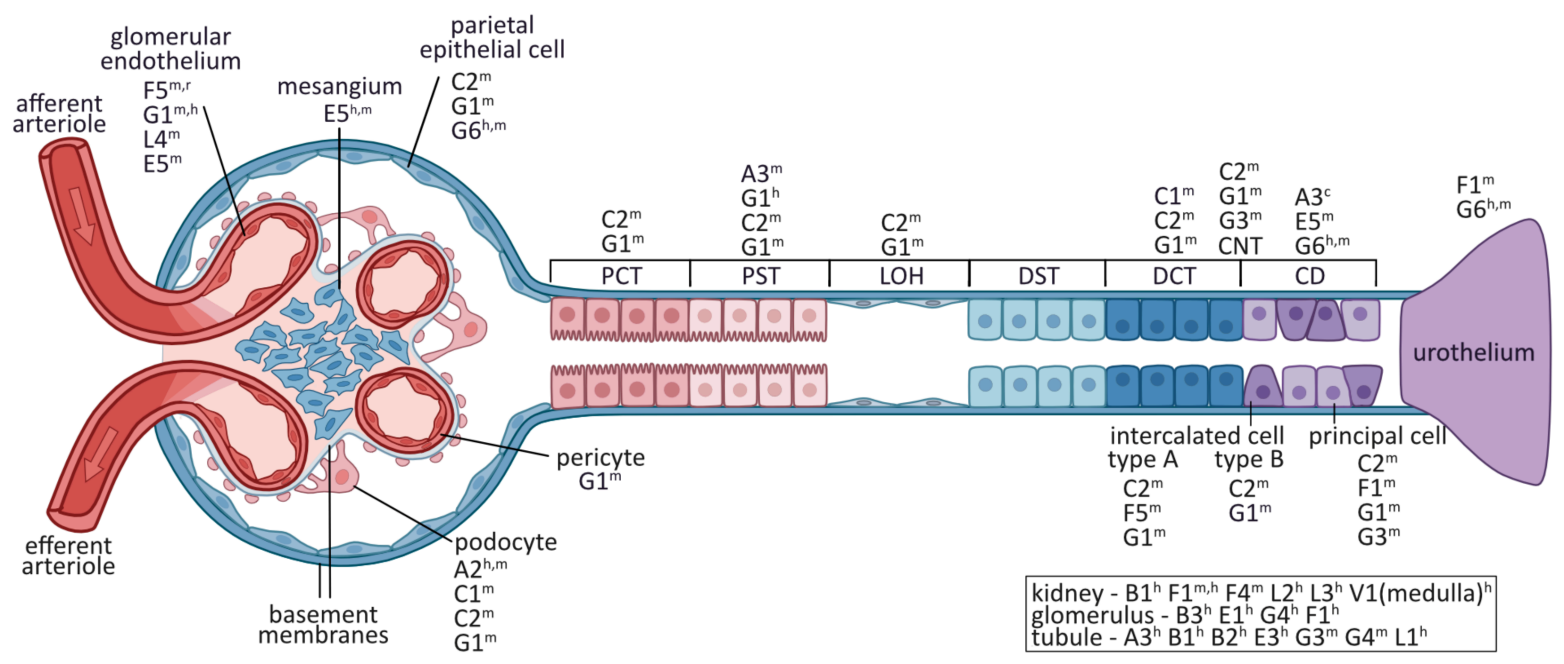
Schematic overview of a metanephric nephron representing known expression patterns of aGPCRs described in Table 1. The aGPCRs are denoted by subfamily and subtype. For expression patterns supported exclusively by snRNA-seq data, only aGPCRs detected in >20% of cells within a given nephron segment or cell type are depicted; patterns reported by other studies are shown regardless of the snRNA-seq detection frequency. The aGPCRs with unknown specific location or broad expression in the kidney, glomerulus, or tubules are listed in the lower-right box. Superscript letters indicate the species in which the expression has been observed: h, human; m, mouse; r, rat; c, canine. PST, proximal straight tubule; PCT, proximal convoluted tubule; LOH, loop of Henle; DCT, distal convoluted tubule; DST, distal straight tubule; CNT, connecting tubule; CD, collecting duct.

The expression of aGPCRs during kidney development and disease had previously been summarized [35]. Currently, the expression pattern of 25 members has been described in the developing, adult, and/or diseased kidney, and for 7 members their functional role in kidney development and/or disease is known (Table 1). In addition, the expression of 12 members is altered in the diseased kidney (Table 2). For 7 members, their functional role or at least a knockout (KO) phenotype in another organ is known. Finally, for 5 members, there are only expression and/or cell-based data available. In Figure 2, we have summarized the currently known renal expression patterns of aGPCRs (Table 1), highlighting those with segment- or cell type-specific expression; broadly expressed aGPCRs are omitted for clarity.

**Table 1:** Renal expression of aGPCRs.

aGPCR	Other Names	Localization in the Kidney	Detection Methods	Refs.
ADGRA1	GPR123	Kidney ^z^	RT-qPCR	[62]
ADGRA2	GPR124, TEM5	Podocytes ^h,m^	IHC, IF	[63]
		Glomeruli ^m^	WB	[63]
		PCT, fibroblasts, pericytes ^m^	snRNA-seq	[64]
ADGRA3	GPR125, PGR21, TEM5L	MDCK cells ^c^	RT-PCR	[65]
		Cortical late DCT and CNT ^h,m^	RT-PCR, immunostaining	Abstract *
		Nephrogenic cortex, E14.5 ^m^; tubular cells, adult ^h^	IHC, ISH (EURExpress, HPA)	[35]
		podocytes, PEC, PCT, PST, LOH, MD, DCT, CNT, PC, IC-A, IC-B, pericytes ^m^	snRNA-seq	[64]
ADGRB1	BAI1	Kidney, PT, DT ^h^	RT-qPCR, IHC	[66]
ADGRB2	BAI2	Tubules, renal pelvis ^h^	IHC, microarray (HPA, Nephroseq)	[35]
ADGRB3	BAI3	Glomerulus ^h^	Microarray (Nephroseq)	[35]
ADGRC1	CELSR1	CD stalks and S-shaped bodies, PT, podocytes, E18.5 ^m^	IHC	[67]
		Podocytes, cultured ^m^	RT-qPCR	[67]
		podocytes, PEC, PCT, PST, LOH, MD, DCT, CNT, PC, IC-A, IC-B, pericytes ^m^	snRNA-seq	[64]
ADGRC2	CELSR2, EGFL2	Podocytes, PEC, PCT, PST, LOH, MD, DCT1/2, CNT, PC, IC-A, IC-B ^m^	snRNA-seq	[64]
		Developing kidney, E14.5 ^m^; medulla ^h^	IHC, ISH, microarray (EURExpress, HPA, Nephroseq)	[35]
ADGRE1	EMR1, F4/80	Glomerulus ^h^	Microarray (Nephroseq)	[35]
ADGRE3	EMR3	Tubular cells ^h^	IHC (HPA)	[35]
ADGRE5	CD97, TM7LN1	Kidney ^m^	NB	[68]
		Kidney ^z^	RT-qPCR	[62]
		Glomerulus, mesangial cells ^h^	IHC	[69]
		Glomerulus, desmin^+^ cells (podocytes, mesangial cells) ^m^	IHC (lacZ reporter mouse), IF	[70]
		Glomerulus, renal pelvis ^h^	Nephroseq	[35]
		CD, E14.5 ^m^	EURExpress	[35]
		Podocytes, PEC, PCT, PST, LOH, DCT, CNT, PC, IC-A, IC-B, endothelial cells ^m^	snRNA-seq	[64]
ADGRF1	GPR110, PGR19	Kidney ^m^	PCR	[71]
		Renal pelvis, ureter ^m^	IHC (lacZ knock-in)	[71]
		Kidney ^h^	RT-qPCR	[72]
		Medullary PC ^m^	snRNA-seq, ISH (RNAscope), RT-qPCR	[64]
		Glomerulus, papillary tips ^h^	Nephroseq	[35]
ADGRF4	GPR115, PGR18	Kidney ^m^	PCR	[71]
ADGRF5	GPR116, Ig-Hepta	IC of the cortical CD ^r^	IF	[73]
		Kidney ^m^	PCR	[71]
		Glomerular and peritubular endothelium (*Pecam1*^+^), IC-A but not *Aqp2*^+^ PC ^m^	ISH (RNAscope)	[64]
		Endothelial cells, IC-A, Podocytes, PEC, PCT, PST, LOH, DCT, CNT, PC, IC-B ^m^	snRNA-seq	[64]
		IC-A, apical (V-ATPase^+^) ^m^	IF, immunogold EM	[33]
		Glomerular microvessels ^m^	mCherry reporter	[74]
		IC ^m^	IHC	[75]
		Glomerular and peritubular capillary endothelium (luminal membrane) ^r^, apical region of IC, some cortical and outer-medullary tubular epithelium ^m,r^	ISH (RNAscope), IF, immunogold EM	[76]
		IC of the CD ^m^	Nephroseq	[77]
ADGRG1	GPR56, BFPP, BPPR, TM7LN4, TM7XN1	*Aqp1*^+^ PT/descending limb and *Aqp2*^+^ CNT/PC ^m^	ISH (RNAscope)	[64]
		Podocytes, PEC, PCT, PST, LOH, MD, DCT, CNT, PC, IC-A, IC-B, pericytes, endothelial cells ^m^	snRNA-seq	[64]
		Renal cortex ^h^; ureteric branches ^m^	Nephroseq, EURExpress	[35]
		GEC ^m,h^	IF, bulk RNA-seq	[78]
ADGRG3	GPR97, PB99, PGR26	PT and DT ^m^	IF	[79]
		PEC, Podocytes, PCT, PST, DCT, CNT, PC, IC-A ^m^	snRNA-seq	[64]
ADGRG4	GPR112, Gm367, PGR17	Glomerulus ^h^	Microarray (Nephroseq)	[35]
		PT ^m^	RNAseq databases	[34]
ADGRG6	GPR126, APG1, Gm222, DREG, PR126, PS1TP2, VIGR	PEC, CD, urothelium, metanephros ^h,m^; ionocytes, pronephros, mesonephros ^z^	ISH (RNAscope)	[36]
		PEC ^m^	snRNA-seq	[64]
ADGRL1	LPHN1, CIRL1, CL1, LEC2	Tubules ^h^	IHC (HPA)	[80]
		Podocytes, PEC, PCT, PST, LOH, DCT, CNT, IC-A ^m^	snRNA-seq	[64]
ADGRL2	LPHN2, CIRL2, CL2, LEC1	Podocytes, PEC, endothelial cells ^m^	snRNA-seq	[64]
		Kidney ^h^	NB	[81,82]
ADGRL3	LPHN3, CIRL3, CL3, LEC3	Kidney ^h^	IHC (HPA)	[80]
ADGRL4	ELTD1, ETL	Glomerular endothelium, adult ^m^	lacZ knock-in reporter	[74]
		Endothelial cells ^m^	IF	[74]
		endothelial cells, podocytes, PEC, PCT, PST, LOH, DCT, CNT, IC-A, IC-B ^m^	snRNA-seq	[64]
ADGRV1	GPR98, FEB4, MASS1, USH2B, USH2C, VLGR1	Podocytes ^m^	snRNA-seq	[64]
		Medulla ^h^	HPA, Nephroseq	[35]

Species: ^h^: human, ^m^: mouse, ^r^: rat, ^z^: zebrafish, ^c^: canine. Nephron segments and cells: CD, collecting duct; CNT, connecting tubule; DCT, distal convoluted tubule; GEC, glomerular endothelial cell; IC-A, intercalated cell type A; IC-B, intercalated cell type B; LOH, loop of Henle; MD, macula densa; PC, principal cell; PCT/PST, proximal convoluted/straight tubule; PEC, parietal epithelial cell; PT, proximal tubule; DT, distal tubule. Methods: EM, electron microscopy; IF, immunofluorescence; IHC, immunohistochemistry; ISH, in situ hybridization; NB, northern blot; RT-PCR, real-time polymerase chain reaction; RT-qPCR, quantitative RT-PCR; snRNA-seq, single-nucleus RNA sequencing; WB, western blot. Resources and models: EURExpress, transcriptome atlas of the mouse embryo; HPA, Human Protein Atlas; MDCK, Madin–Darby canine kidney cells; Nephroseq, renal transcriptome database. Refs.: References. * Conference abstract (doi:10.1152/physiol.2025.40.S1.1215); not peer reviewed.

**Table 2:** Loss-of-function phenotypes and human disease associations of aGPCRs in the kidney.

aGPCR	Model	Renal Loss-of-Function Phenotype	Association with Human Kidney Disease	Refs.
ADGRA2	Mouse, podocyte-specific KO	Exacerbated DKD	DKD and renal fibrosis: reduced podocyte ADGRA2 expression in DKD; expression in activated (*Tcf21*^+^/α-SMA^+^) fibroblasts in CKD	[63,83]
	Human			
ADGRA3	MDCK 3D culture, CRISPR/Cas9 KO and knockdown	Multi-lumen cysts with misoriented mitotic spindles	—	[65]
ADGRB1	Human; mouse RCC model	—	RCC: reduced expression, inversely correlated with tumor microvessel density; *Adgrb1* gene transfer reduces tumor growth and angiogenesis in mice	[66,84]
ADGRC1	Mouse, *Celsr1*^Crsh^ and global KO	Smaller, less complex ureteric tree (fewer tips, segments, and branch points); cortical tubular dilation; altered orientation of tubular mitoses at E17.5	—	[85]
	Human	—	*ADGRC1* variants with spina bifida: increased frequency of renal tract malformations (agenesis, hydronephrosis, hydroureter) versus reference populations	
ADGRC3	Zebrafish, *adgrc3* knockdown	Urinary-tract malformation, rescued by WT human ADGRC3 mRNA	—	[86]
	Human	—	Bi-allelic *ADGRC3* variants implicated in CAKUT	
ADGRE1	Human	—	Diabetic and lupus nephritis: upregulated	[35]
ADGRE5	Mouse, germline KO	No overt baseline renal phenotype reported	—	[70]
	Human	—	Clear cell RCC, diabetic and lupus nephritis, renal vasculitis, and IgA nephropathy: upregulated	[35]
ADGRF1	Mouse, germline KO lacZ knock-in	No overt baseline renal phenotype reported	—	[71]
ADGRF4	Mouse, germline KO lacZ knock-in	No overt baseline renal phenotype reported	—	[71]
ADGRF5	Mouse, global KO	No overt cardiovascular or renal phenotype	—	[74]
	Mouse, *Ksp*-Cre conditional KO	Apical accumulation of V-ATPase in IC-A→ excessive urinary acidification and metabolic alkalosis (renal acid secretion inhibited)	—	[33]
	Mouse, *Adgrf5*/*Adgrl4* double KO	50% perinatal lethality; glomerular thrombotic microangiopathy; proteinuria	—	[74]
ADGRG1	Mouse, *Adgrg1* KO	No overt baseline phenotype, but protective in streptozotocin-induced diabetes: ADGRG1 suppresses eNOS via PKA/KLF4 in glomerular endothelium	—	[78]
	Human	—	DKD: elevated in glomeruli, inversely correlated with renal function	
ADGRG2	Human	—	Diabetic nephropathy, CKD, and lupus nephritis: upregulated	[35]
ADGRG3	Mouse, *Adgrg3* KO	Protective in AKI (less injury and inflammation after ischemia–reperfusion and cisplatin; Sema3A/HuR); reduced interstitial fibrosis in hypertensive nephropathy	Acute tubular necrosis: upregulated	[78,79]
	Human			
ADGRG6	Human	—	Altered expression in disease: increased in PECs in FSGS; AKI; CKD	[87]
ADGRL2	Human	—	Lupus, diabetic, and IgA nephropathy: upregulated	[35]
ADGRL4	Mouse, *Adgrl4* KO	No overt cardiovascular or renal phenotype	—	[74]
	Mouse, *Adgrl4*/*Adgrf5* double KO	50% perinatal lethality; glomerular thrombotic microangiopathy; proteinuria	—	[74]
	Human	—	Clear cell RCC: ADGRL4^+^ tubule cells identified as a highly aggressive population (single-cell and spatial transcriptomics)	[88]

Abbreviations: AKI, acute kidney injury; CAKUT, congenital anomalies of the kidney and urinary tract; CKD, chronic kidney disease; DKD, diabetic kidney disease; eNOS, endothelial nitric oxide synthase; FSGS, focal segmental glomerulosclerosis; KO, knockout; KLF4, Krüppel-like factor 4; MDCK, Madin–Darby canine kidney; PKA, Protein kinase A; PECs, parietal epithelial cells; RCC, renal cell carcinoma; WT, wild-type; α-SMA, α-smooth muscle actin. Refs.: References.

### 3.1. aGPCRs with a Well-Defined Role in Renal Development and/or Physiology

aGPCRs (the ADGR family) contribute at several levels to kidney development and function, from UB branching and tubular morphogenesis to vascular integrity and acid–base regulation.

#### 3.1.1. CELSR1/ADGRC1 and Planar Cell Polarity in Nephrogenesis

**Figure 3:**
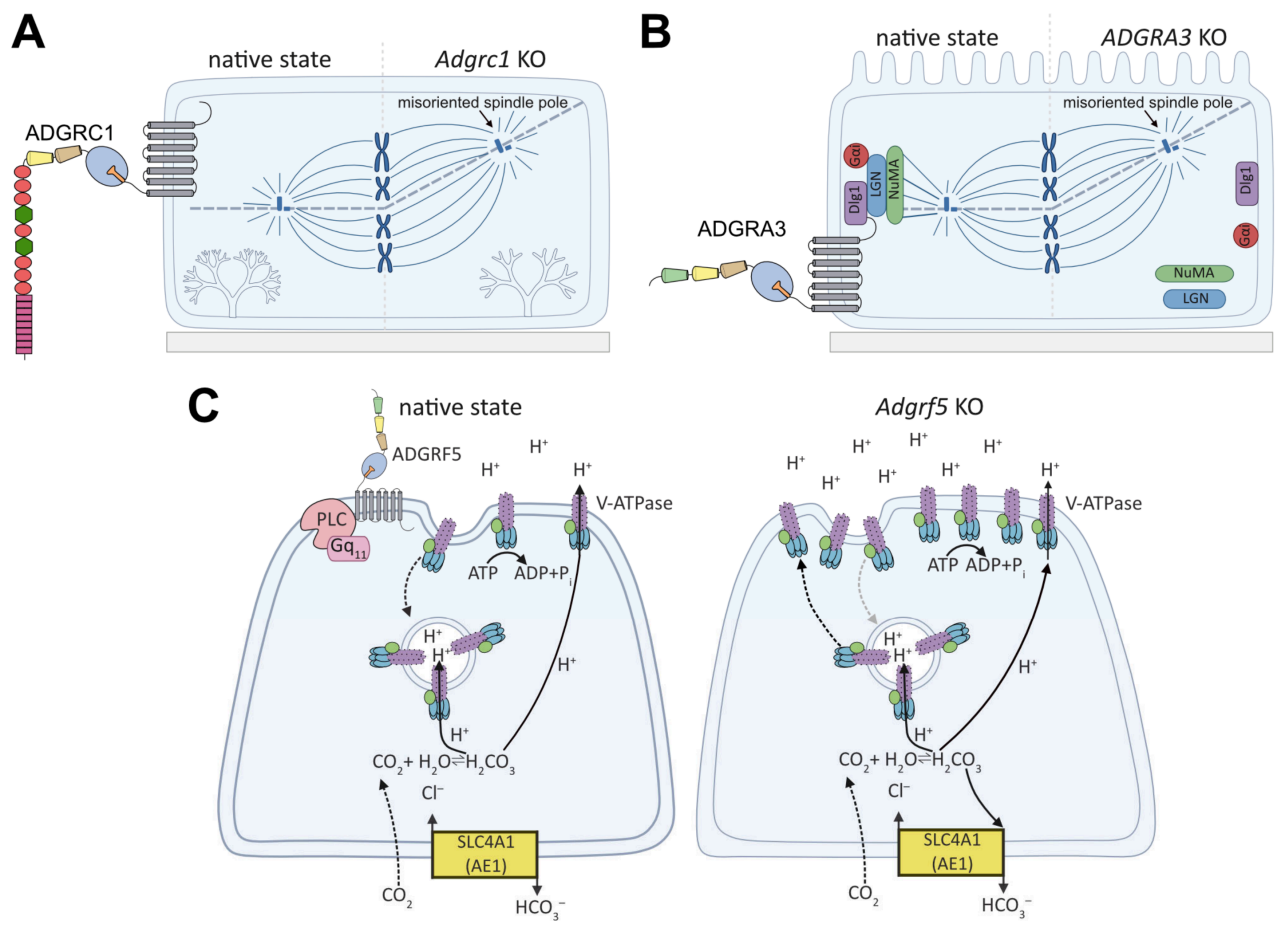
aGPCRs in renal development and physiology. (**A**) Model of CELSR1/ADGRC1 function in planar cell polarity (PCP) during nephrogenesis. In developing kidneys, ADGRC1 localizes to the membranes of ureteric and tubular epithelial cells and contributes to PCP signaling. Proper ADGRC1 function supports correct spindle orientation during ureteric tree branching and tubular morphogenesis. In *Adgrc1* knockout (KO) kidneys, loss of PCP signaling caused misoriented spindle poles with respect to the basal lamina. *Adgrc1* KO kidneys presented with simplified, hypoplastic ureteric trees with reduced branching. (**B**) Illustration of ADGRA3/GPR125 function in Madin–Darby canine kidney (MDCK) cells. In the native state (**left**), DLG1 localizes ADGRA3 to the basolateral membrane, where they associate with other polarity regulators, LGN and NuMA, to ensure proper mitotic spindle orientation. In *Adgra3* KO MDCK cells (**right**), loss of ADGRA3 disrupted the mitotic spindle orientation machinery and resulted in misoriented spindle poles. (**C**) Schematic representation of GPR116/ADGRF5 function in type A intercalated cells (IC-As) in wild-type (WT, (**left**)) and renal tubule-specific *Adgrf5* KO mice (**right**), adapted from Zaidman et al. [33]. In WT mice, ADGRF5 localizes to the apical membrane of IC-As, coupling most likely to Gα_q/11_, limiting apical V-ATPase surface density and proton secretion into the tubular lumen, regulating urinary pH. In *Adgrf5* KO (**right**), V-ATPase translocation is misregulated, leading to increased recruitment of V-ATPase-containing vesicles to the apical membrane, enhanced proton secretion, and a lower urinary pH.

CELSR1/ADGRC1 is a well-characterized component of the PCP machinery in the developing mammalian kidney (Figure 3A). In *Adgrc1* KO mouse embryos, analysis at E13.5 revealed rudimentary UB trees with reduced branching complexity—quantified as fewer tips, segments, and branching points—indicating a critical role for ADGRC1 in early branching morphogenesis of the collecting system [85]. Compound heterozygous mutations of *Adgrc1* and *Vangl2*, another core PCP gene, exacerbated these UB branching defects, including a disturbed caudal/rostral branch ratio due to a selective reduction in caudal branches, highlighting genetic interaction within the PCP pathway during renal tract patterning [85].

In addition to its role in UB branching, *Adgrc1* mutants exhibit defects during late nephrogenesis. By E17.5, *Adgrc1* loss led to global kidney growth retardation and dilated cortical tubules. Quantitative assessment of phospho-histone H3–positive mitoses revealed that tubular cell divisions are less frequently aligned with the longitudinal tubular axis and more often oriented at oblique angles than in controls [85]. This association between altered spindle orientation and tubule dilation is consistent with the idea that ADGRC1 helps maintain proper tubular diameter via PCP-dependent control of oriented cell division, although it does not by itself prove a direct mechanistic link.

Clinically, in human fetuses and neonates carrying heterozygous *ADGRC1* variants and exhibiting spina bifida, renal tract malformations, such as unilateral renal agenesis, hydronephrosis, and hydroureter, are reported more frequently than expected [85]. The co-occurrence of ADGRC1 mutations and renal disease/malformations suggests that ADGRC1 might also play a role in humans; however, the available data do not allow firm conclusions about penetrance or causality.

#### 3.1.2. GPR125/ADGRA3 in Renal Epithelial Polarity and Cystogenesis

GPR125/ADGRA3 is an aGPCR implicated in PCP and epithelial organization. In zebrafish, Adgra3 acts as a modulator of non-canonical Wnt/PCP signaling, recruiting disheveled (Dvl) to the plasma membrane and clustering it into discrete membrane subdomains during convergence-extension movements; excess Adgra3 impairs these movements and the underlying cell polarities, whereas reduced Adgra3 function exacerbates the defects of PCP mutants [89]. Although this was established in gastrulating embryos rather than in the developing kidney, the PCP machinery involved is the same that governs pronephric tubule elongation, identifying ADGRA3 as a PCP-competent receptor whose renal relevance remains to be tested directly.

Kidney-relevant evidence comes from mammalian epithelial models. In Madin–Darby canine kidney (MDCK) cells, ADGRA3 underwent cis-autoproteolysis at an atypical GPS within the GAIN domain, and CRISPR-mediated deletion of *ADGRA3* in three-dimensional Matrigel^TM^ culture led to abnormal multi-lumen cysts, whereas control cells predominantly formed single-lumen cysts [65]. In these cyst models, ADGRA3 localized basolaterally, and its correct localization depended on its C-terminal PDZ-binding motif and its interaction with the polarity scaffolding protein “discs large homolog 1” (DLG1); *DLG1* knockdown reduced basolateral ADGRA3 and produced similar cystic defects. Cysts derived from ADGRA3-deficient cells showed a broader distribution of mitotic spindle angles, indicating less constrained spindle orientation, and co-immunoprecipitation experiments demonstrated preferential association of ADGRA3 with Gα_i2_ and Gα_i3_. On this basis, a DLG1–ADGRA3–Gα_i_ axis has been proposed to contribute to spindle orientation via the NuMA–LGN–Gα_i_ complex in renal epithelia (Figure 3B). At present, this mechanism is well supported in cyst and cell-culture models, whereas direct in vivo data from kidney tubules are not yet available.

#### 3.1.3. GPR116/ADGRF5 in the Glomerular Endothelium

GPR116/ADGRF5 protein is present on GECs lining the capillary walls [74,76]. The endothelial aGPCRs ADGRF5 and ELTD1/ADGRL4 have been interrogated in double-KO mice to assess their combined cardiovascular and renal roles. Single deletion of either *Adgrf5* (targeting exon 8) or *Adgrl4* (targeting exon 5) caused neither overt renal pathology nor gross cardiovascular malformation [74]. Mice lacking both *Adgrf5* and *Adgrl4*, however, exhibited malformations of the aortic arch arteries and cardiac outflow tract, and approximately 50% died perinatally [74]. Surviving double-KO mice developed renal thrombotic microangiopathy with schistocytes in glomerular capillaries, narrowed capillary lumina, proteinuria, elevated plasma urea, and reduced lifespan. Ultrastructural analysis revealed loss of endothelial fenestrations and podocyte foot process effacement, consistent with a compromised GFB. Notably, neither endothelial cell-specific nor neural crest-specific double KO recapitulated the renal phenotype, suggesting that ADGRF5/ADGRL4 expression in other cell types, or additional developmental roles, contributed to the kidney pathology. Because the vascular and renal abnormalities co-occurred with major cardiac malformations in this model, it remains difficult to distinguish direct renal effects from systemic hemodynamic or hematological consequences. A subsequent study has demonstrated that ADGRF5 resides on the luminal surface and fenestrae of GECs in the adult mouse kidney. In-depth analysis of another *Adgrf5* KO mouse (targeting exon 2) revealed impaired renal function, albuminuria, and glomerular hypertrophy [76]. Complex ultrastructural changes included GBM splitting, thickening and duplication, GEC detachment, diaphragmed fenestrations with reduced open fenestrated area, mesangial interposition, and increased podocyte foot process width. Together, these findings support a model in which ADGRF5 at the blood–urine interface contributes to GFB integrity and to the fenestrated architecture required for selective filtration. The observation of different phenotypes in the two *Adgrf5* KO mouse models might be due to the different *Adgrf5* gene targeting strategies. According to UniProt and NCBI Gene annotations, exon 2 codes for the start codon and signal peptide but no functional domain, while exon 8 encodes part of the immunoglobulin-like domain after the SEA domain of the NTF. Hence, *Adgrf5* KO mice targeting exon 8 might still express a functional truncated NTF up to the SEA domain, potentially compensating for the glomerular phenotype. Furthermore, splicing events might play a role, as 105 different *Adgrf5* transcripts encoded by 79 exons have been described [90].

#### 3.1.4. ADGRF5 in Intercalated Cells and Renal Acid–Base Regulation

Beyond the glomerular endothelium, ADGRF5 has also been localized to the acid-secreting IC-A of the cortical CD, first in rat [73] and subsequently in mouse [75] utilizing antibody-based detection [33,75]. Mice with kidney tubule-specific *Adgrf5* deletion (Ksp1.3-Cre) produced more acidic urine and exhibited mild alkalemia compared with littermate controls—a pattern the authors termed “renal tubular alkalosis” [33]. Light microscopy did not reveal major structural abnormalities in the kidneys, and blood pressure was not significantly altered, suggesting that the primary defect under baseline conditions is functional dysregulation of acid secretion rather than gross developmental or hemodynamic change. At the cellular level, immunogold labeling and morphometric analysis demonstrated an increased apical surface density of vacuolar-type H^+^-ATPase (V-ATPase) in IC-As from *Adgrf5* KO mice, consistent with enhanced luminal availability of proton pumps in the absence of receptor signaling. In split-open CD preparations, application of a synthetic tethered-agonist peptide (p16) that activates ADGRF5 reduced proton extrusion in ICs and increased intracellular calcium specifically in V-ATPase–positive IC-As, whereas neighboring principal cells showed no comparable response. Together, these data support a model in which ADGRF5 acts as an inhibitory modulator of V-ATPase-dependent proton secretion in IC-As, likely through calcium-dependent signaling (Figure 3C). The endogenous ligand(s), and the extent to which this pathway is engaged under physiological or pathophysiological acid–base challenges, remain to be defined.

In summary, current data implicate ADGRC1 in UB branching and tubular morphogenesis, ADGRA3 in epithelial polarity and spindle orientation in renal cyst models, and ADGRF5 in both glomerular–vascular integrity and IC-A–mediated acid–base regulation. Notably, these receptors converge on a common set of themes—oriented cell division, epithelial polarity, and the structural integrity of specialized renal barriers—reinforcing the view that aGPCRs couple adhesive and mechanical context to renal form and function.

### 3.2. aGPCRs with a Disease Link and/or an Examined KO Model but Unknown Renal Function

Most of the aGPCRs expressed in the kidney have been characterized in other tissues/organs. Thus, while their renal function remains elusive, this information, e.g., regarding established ligands (Table 3), signaling pathways, and cell behavior, can possibly be utilized, combined with the renal expression data, to hypothesize their role in the kidney. Therefore, we have analyzed whether known ligands of renal aGPCRs are known to be present in the kidney and whether ligand-mediated signaling pathways are known to play a role in kidney development, physiology, and/or disease. Based on this analysis, we discuss potential roles of renal aGPCRs in the following sub-chapters.

#### 3.2.1. GPR126/ADGRG6: N-Cadherin and Basement Membrane Sensing

*Adgrg6* is expressed in parietal epithelial cells (PECs), CD, and urothelium, and its expression is modulated in a number of different kidney diseases and disease models [36]. To date, no studies are available investigating a renal phenotype upon *Adgrg6* deletion. Straight *Adgrg6* KO mice are embryonic lethal, and a renal phenotype in available adult *adgrg6* zebrafish mutants has not yet been described. *adgrg6* zebrafish mutants exhibit defects in maintaining cell–cell adhesion in cardiomyocytes, with N-cadherin mislocalization [181]. Notably, N-cadherin is mainly expressed in PTs [182], the segment in which *Adgrg6* is expressed only during disease [87]. In experimental and human AKI, N-cadherin is depleted from PTs [183]. Potentially, *Adgrg6* upregulation is a mechanism to stabilize cell–cell adhesion.

While ligand-induced signaling of aGPCRs is still poorly understood, several ligands of ADGRG6 have been identified: type IV collagen, laminin-211, and collagen VI [152] which, together with mechanical load, trigger Gα_s_- and Gα_i_-mediated cAMP signaling [153,154]. Core structural components of basement membranes consist of laminin, type IV collagen, nidogen, and heparan sulfate proteoglycan. However, basement membranes differ significantly in the types of laminin and type IV collagen [184]. For example, the mature GBM consists of collagen α3α4α5 (IV) and laminin-521 [185]. In contrast, the basement membrane of Bowman’s capsule, the DTs, and CDs—where *Adgrg6* is expressed in PECs and tubular cells—contains α5α5α6 collagen IV alongside the ubiquitous α1α1α2 collagen IV [186,187]. Thus, ADGRG6 might act as a sensor for the mechanical state of the basement membranes to detect via α5α5α6 collagen IV mechanical load/stretch of the Bowman’s capsule and the CDs. This might also explain the upregulation of *Adgrg6* in kidney disease [87]. Future studies utilizing kidney- and PEC-specific *Adgrg6* KO mice in renal injury models will reveal whether ADGRG6 might be a potential therapeutic target.

In addition, progesterone and 17-hydroxyprogesterone were identified as activators, both acting through Gα_i_ [157]. Although the reproducibility of steroid binding across adhesion GPCRs is debated [188]. Progesterone is known to act on the CD by competing with aldosterone at the nuclear mineralocorticoid receptor. While nuclear receptors are generally slow in transmitting signals, as their main effect is changing gene transcription and protein synthesis, which usually takes minutes to hours, cell surface receptors are generally faster because they trigger pre-existing intracellular signaling pathways, so responses can occur in seconds to minutes. Thus, *Adgrg6*, which is also expressed in CDs, provides a potentially faster response mechanism.

#### 3.2.2. CD97/ADGRE5: Primary Cilium

*Adgre5* is expressed in the CD system of E14.5 mouse embryos (EURExpress, sections 9–11, 18–21) and in podocytes and mesangial cells in adult mice. Yet, *Adgre5* KO mice do not exhibit a kidney phenotype [70]. ADGRE5 is also abundant on granulocytes, monocytes, and activated lymphocytes, mediating leukocyte adhesion and migration, with the complement regulator CD55 as its best-characterized ligand [118,120]. CD55 is expressed on renal endothelium, podocytes, and tubular epithelium as part of the kidney’s intrinsic complement defense. The renal *ADGRE5* signal is upregulated in lupus nephritis, vasculitis, and IgA nephropathy [35], which might be due to the infiltrating CD97-expressing leukocytes. In terms of potential renal functions of ADGRE5, it has been described that outside the immune system, ADGRE5 influences cell–cell adhesion, adherens junctions, migration, and invasion in epithelia and tumors [117,189,190,191,192]. Mechanical forces induce protein kinase C- and D-dependent phosphorylation of ADGRE5, disrupting its interaction with the PDZ domain of the scaffold DLG1, resulting in cytoskeleton remodeling [125]. This interaction is noteworthy as DLG1 has been implicated in kidney development. DLG1 regulates primary cilia length and kidney morphology [193]. Conditional *Dlg1* KO mice exhibit ciliary elongation, cystogenesis, and impaired ciliary targeting of polycystin-2 [194]. Based on its expression, *Adgre5* might play a role in ciliogenesis of the CD, as the primary cilium is especially important in the tubular epithelium of the kidney, mainly the PT and DT as well as the CD [195,196]. Notably, also GPR125/ADGRA3 has been reported to interact with Dlg1. In MDCK cells, ADGRA3 and DLG1 are both recruited to their basolateral domain of the plasma membrane, and knockdown of each protein resulted in cystogenesis [65]. Thus, *Adgra3* might be able to compensate for the loss of *Adgre5*.

#### 3.2.3. BAI1/ADGRB1: Efferocytosis by the Tubular Epithelium

Renal *Adgrb1* is well characterized, with expression in PTs and DTs [66]. *Adgrb1* KO mice exhibit brain and behavioral phenotypes [197]. Yet, a kidney phenotype has so far not been described. KO studies in hippocampal neuron-glia cultures indicated that BAI1 is required for neuronal development, relying on its interaction with reticulon-4 receptors (RTN4Rs) [100,111]. A correlation to kidney development is not apparent in this case. ADGRB1 is reported to recognize phosphatidylserine on apoptotic cells through its thrombospondin repeats, driving engulfment of apoptotic cells via the ELMO/DOCK180/Rac signaling module [98]. Notably, PT cells are themselves semi-professional phagocytes utilizing the “kidney injury molecule-1/T cell IgG and mucin containing 1” (KIM-1/TIM-1) receptor recognizing phosphatidylserine on apoptotic cells, inducing the binding and uptake of dead cells from the kidney tubule lumen in AKI [198]. Thus, Adgrb1 might enhance the efferocytotic capacity of the tubules or might be involved in KIM-1/TIM-1 signaling.

Overexpression studies indicate that *Adgrb1* acts as an anti-angiogenic receptor that suppresses endothelial proliferation and vessel formation via α_v_β_5_ integrin [199].

#### 3.2.4. GPR110/ADGRF1: Metabolism

*Adgrf1* is highly expressed in the renal pelvis and ureter urothelium [71]. Yet, *Adgrf1* KO mice exhibit no obvious phenotype. Interestingly, there is accumulating evidence that ADGRF1 expression is required to protect from disease. Hepatocyte-specific *Adgrf1* KO protects against “metabolic dysfunction-associated steatohepatitis (MASH)” in female, but not male mice, and the *ADGRF1* variant rs937057 T > C is associated with a higher prevalence of metabolic dysfunction-associated steatotic liver disease in women. Mechanistically, activation of ADGRF1 signaling results in suppressed expression of hepatic estrogen receptor alpha (*Esr1*) [200]. In addition, *Adgrf1* expression is associated with hepatic de novo lipogenesis by regulating Stearoyl-CoA desaturase 1 (Scd1) [201]. This might explain why, under physiological conditions, an *Adgrf1* KO does not exhibit an obvious phenotype. Future studies of renal injury models in *Adgrf1* KO mice might reveal whether ADGRF1 acts in a protective manner.

#### 3.2.5. LPHN2/ADGRL2: Endothelial Shear Sensing Under Hyperfiltration

Expression of *ADGRL2*/*Adgrl2* in the kidney is poorly characterized. Northern and western blot analyses revealed expression in the adult rat and human kidney [81,82,202]. Single-nucleus RNA sequencing (snRNA-seq) suggested expression in podocytes, PECs. and endothelial cells. ADGRL2 is a fluid-shear-stress sensor of vascular endothelium acting upstream of PECAM-1 and is required for flow-dependent angiogenesis and arterial remodeling [161]. It is localized at cell–matrix adhesions and signals through cAMP and Rap1, while restraining nuclear translocation of Yes-associated protein (YAP) and transcriptional coactivator with PDZ-binding motif (TAZ) [158]. *adgrl2* KO leaves endothelial cells in zebrafish abnormally stretched with defective intercellular junctions [158,161]. Shear stress plays a major role in several compartments of the kidney. Besides the blood vessels in the kidney, shear stress is important in the renal tubules, especially the PT, where tubular fluid flow acts on the apical surface of epithelial cells, regulating transport, protein uptake, barrier function, and metabolism [203,204]. In addition, shear stress affects podocytes and PECs when urinary ultrafiltrate flows between the podocytes into Bowman’s space [205]. Consequently, ADGRL2 could have several functions in the kidney. However, before further speculating about a potential function of ADGRL2 in the kidney, it appears important to validate ADGRL2 protein expression in the kidney, determine the expressing cell type, and assess the subcellular localization.

#### 3.2.6. GPR64/ADGRG2: Apical Fluid and pH Handling in the Collecting Duct

Nephroseq data suggest that *ADGRG2* is expressed in a number of kidney diseases but is absent during kidney development or in the healthy kidney. Previously, it has been shown that truncating mutations of *ADGRG2* can cause X-Linked Congenital Bilateral Absence of Vas Deferens [206,207]. ADGRG2 occupies the apical membrane of the non-ciliated, microvillous cells of the efferent ductules of the male reproductive system [208], where around 90% of luminal fluid is reabsorbed [209]. *Adgrg2* KO in mice resulted in fluid accumulation and obstructive infertility [210]. Notably, “Cystic Fibrosis Transmembrane Conductance Regulator (CFTR)” mutations can cause X-Linked Congenital Bilateral Absence of the Vas Deferens [211]. ADGRG2 is constitutively active and couples through Gα_q_. It forms a complex with CFTR via β-arrestin-1 [212]. This complex is located apically in the cells and is essential for fluid reabsorption. Loss of any component reduces CFTR current and disturbs luminal pH [212]. While the efferent ductules derive from the mesonephric tubules, the UB and hence the whole collecting system arise from the mesonephric (Wolffian) duct itself, as do the epididymal duct and vas deferens [213]. Notably, epididymal clear cells as well as IC-A are recognized counterparts, both concentrating V-ATPase at the apical membrane to acidify the lumen [33]. This suggests that ADGRG2 might contribute to pH handling in renal IC-A, as described above for ADGRF5. Notably, aldosterone enhances V-ATPase trafficking in the ICs of CD, facilitating proton secretion and acid–base homeostasis. In contrast, ADGRF5 acts to decrease surface accumulation of V-ATPase [33]. In addition, aldosterone also promotes inflammation and fibrosis in the kidney by activating the mineralocorticoid receptor in other cellular compartments, including podocytes, mesangial cells, epithelial cells, and myeloid cells [214]. Considering that dehydroepiandrosterone (DHEA) acutely potentiates the whole-cell chloride current in primary non-ciliated efferent ductule cells, an effect abolished by *Adgrg2* deletion or by CFTR inhibition, and that deoxycorticosterone antagonizes it [215], ADGRG2 appears to be downstream of the mineralocorticoid pathway. Whether ADGRG2 contributes during diabetic nephropathy, CKD, and lupus nephritis [35], all conditions with disturbed tubular fluid and acid handling and increased *ADGRG2* expression, to enhance apical fluid and pH handling or promote inflammation and fibrosis remains elusive.

#### 3.2.7. CELSR3/ADGRC3: Primary Urinary Tract Morphogenesis

Until recently, ADGRC3 had not been considered to be relevant in kidney development or disease, as no expression was observed in the kidney [35]. However, in 2024, biallelic variants in *ADGRC3* were described in humans associated with central nervous system and urinary tract anomalies [86]. The patients with or without parallel central nervous system (CNS) phenotype exhibited duplicated collecting system, ectopic kidney, multicystic dysplastic kidney, vesico-ureteric reflux, hydronephrosis, obstructive uropathies, or irregular bladder wall. While mutations in CNS-affected patients reside in NTF as well as CTF coding regions, all variants identified in individuals with a renal phenotype resided within the NTF. Thus, *ADGRC3* might not be expressed in the kidney, but its NTF might affect kidney development in a paracrine manner. The authors of this study performed a detailed *ADGRC3* analysis in the human embryonic metanephros and the zebrafish embryonic pronephros. Expression was detected by antibody staining in human medullary CDs, UB branch stalks, Bowman capsule of immature glomeruli, PTs, uncondensed metanephric mesenchyme, and the urogenital sinus tube, the precursor of the bladder urothelium. Notably, it is unclear if the utilized antibodies have been validated with KO tissues. According to the manufacturer, the antibody detects an EGF-like domain in the NTF. To validate the role of *adgrc3* in renal development, the authors performed morpholino (MO)-mediated knockdown as well as F0 CRISPR KO studies in zebrafish but no expression analysis. The observed MO-induced renal phenotype at 3 days post-fertilization was significant dilatation of the glomerulus and a reduced size of the neck segments. While these data indicated a role of *adgrc3*, it is currently difficult to develop a hypothesis, except that ADGRC3 is similar to ADGRC1, known to be involved in PCP. ADGRC3 is required for ependymal ciliogenesis for proper beating and fluid flow and axon guidance via PCP signaling [216,217].

### 3.3. aGPCRs for Which Only Their Expression Patterns Are Known

For some aGPCRs expressed in the kidney, there is neither a disease association described nor is an analysis available of the kidney in the respective KO animal model.

#### 3.3.1. CELSR2/ADGRC2: Planar Polarity in Partnership with CELSR1

*Adgrc2* and *Adgrc1* are co-expressed in the developing kidney, whereby *ADGRC2* is more broadly expressed [35,218]. ADGRC2 and ADGRC1 are closely related aGPCRs, but they have diverged in their primary biological roles and adhesive properties. Both are core components of the PCP pathway, yet ADGRC1 is the dominant PCP effector in epithelia (e.g., skin, neural tube), whereas ADGRC2 is more specialized for neuronal development, ciliogenesis, and non-epithelial PCP contexts [219,220]. Notably, *Adgrc1* KO abolished PCP protein asymmetry and hair follicle polarization, whereby the phenotype was only minimally enhanced by additional *Adgrc2* KO. The most apparent difference is the more specialized role of ADGRC2 in cilia. While ependymal cilia are motile 9+2 organelles that generate cerebrospinal fluid flow [221], renal cell primary cilia are non-motile (9+0) sensory antennae that transduce mechanical and chemical signals [195,222]. Primary cilia and motile cilia share core assembly machineries (basal bodies, transition zone, intraflagellar transport) but differ in axonemal architecture, number per cell, motility, and principal physiological role. Notably, *ADGRC2* is a putative gene in Joubert syndrome [223], a genetically and clinically heterogeneous group of ciliopathies with the variable feature of fibrocystic disease of the kidneys. However, in the one described 8-year-old female patient carrying an *ADGRC2* mutation, kidney-related serum chemistries and abdominal ultrasonography were normal, arguing against a role in renal ciliogenesis. A genome-wide association study of American Indians identified an *ADGRC2* mutation, proposing *ADGRC2* as a potential regulator of energy metabolism [224], yet no data regarding the kidney were provided. Thus, the current available information is not sufficient to draw any conclusions.

#### 3.3.2. GPR112/ADGRG4: An Apical Sensor at the Proximal Tubule Brush Border

In non-renal tissues, *ADGRG4* marks intestinal enterochromaffin cells [225]. ADGRG4 carries a dimerizing N-terminal pentraxin-like domain, followed by an exceptionally long mucin-like serine and threonine stalk, and couples to Gα_s_ and cAMP. The dimerization itself is established (resolved by X-ray crystallography) and shown to be conserved across mammalian ADGRG4 [226] whereas force sensing via the pentraxin-like domain is proposed based on structural grounds for enterochromaffin and Paneth cells of the small intestine [44]. Given the reported PT RNA expression of *Adgrg4* [34] and since PT microvilli are established flow sensors whose bending modulates sodium reabsorption [227,228] ADGRG4 appears to be a reasonable candidate as a flow sensor. Determining whether ADGRG4 is expressed in the apical surface of PT cells would further substantiate this hypothesis.

#### 3.3.3. Podocytes vs. Neurons

LPHN1/ADGRL1, LPHN3/ADGRL3, and BAI3/ADGRB3, for which in the kidney only their glomerular and tubular expression is reported [35] are known as synaptic adhesion receptors. ADGRL members are known interactors of teneurins, fibronectin leucine-rich transmembrane (FLRT) family members, and/or neurexins [229]. Also, ADGRB3 (BAI3) has been implicated in synapse formation, as it interacts with high affinity with C1q-like molecules [107]. All these interactors are single-span transmembrane molecules that have roles in axon guidance, neuronal connectivity, and synapse formation [44]. Notably, podocytes and neuronal synapses share striking molecular and functional similarities. Podocytes express many “synaptic” proteins, organize their cytoskeleton in neuron-like ways, and contain neuron-like functional synaptic vesicles [230,231]. The proteins classically associated with synapses are functionally important for the GFB [230,232]. Synaptopodin, a key organizer of dendritic spine structure, is critical for foot process stability. Dynamin, synaptojanin 1, and endophilins, core components of clathrin-mediated endocytosis (CME), are important regulators of the GFB. Selective KO of the respective genes causes foot process effacement, underdeveloped GFB, and/or severe proteinuria leading to kidney failure. Thus, we hypothesize that ADGRL1, ADGRL3, and ADGRB3 play a role in GFB function and/or homeostasis.

### 3.4. aGPCRs with a Very Weak Link to Renal Function

VLGR1/ADGRV1: Based on databases assessing RNA expression, bulk-array ADGRV1 expression was reported in the human medulla [35]. However, ADGRV1 is expressed almost ubiquitously, with strongest expression in the developing and mature nervous system [233,234,235]. *ADGRV1* loss of function causes Usher syndrome type 2C in humans, whereas loss-of-function mutations in the mouse ortholog *Adgrv1* caused audiogenic seizures with sensory deficits [236,237,238,239]. Despite the fact that Usher cohorts are thoroughly phenotyped, no renal disease features were detected. Similarly, no kidney phenotype was reported for *Adgrv1* KO mice. Recently, it has been shown that ADGRV1 controls glutamate homeostasis in hippocampal astrocytes supporting neurons [240]. Considering that podocytes possess glutamate-containing vesicular structures that undergo spontaneous and regulated exoendocytosis [231,241], ADGRV1 might play a role in this process.

GPR115/ADGRF4: Similar to ADGRV1, ADGRF4 expression was described for many tissues. It is strongly expressed in the skin, and PCR (polymerase chain reaction) gave a weak signal in the kidney as well as in the testis, ovary, epididymis, and thymus. Notably, *Adgrf4* KO in mice did not affect development or fertility [71].

GPR123/ADGRA1: In our last review, we stated that the analysis of aGPCRs during zebrafish development suggests that *adgra1l* (*gpr123l*) is also expressed in the adult zebrafish kidney [35]. However, revisiting the original publication showed that *adgra1* is not expressed, and the relative expression (fold change) of *adgra1l* is around 0.8 [62]. Notably, fold changes are relative to expression at 21 days post-fertilization and thus difficult to interpret. Main expression for *adgra1* and *adgra1l* was muscle (~2-fold) and brain/eye (2- to 2.5-fold), respectively. The subsequent analysis of *Adgra1* KO mice confirmed high brain expression with no detection in the kidney [242].

EMR1/ADGRE1 and EMR3/ADGRE3: The Nephroseq database suggests that *ADGRE1* is enriched in the glomerulus. In addition, increased expression of these two aGPCRs is associated with lupus nephritis and diabetic nephropathy [35]. Based on the known functions of these receptors, ADGRE1 is an eosinophil-specific receptor [243] and EMR3 tracks granulocyte maturation [244]. Their expression in kidney disease is most likely due to acute interstitial nephritis, the classic eosinophil-associated renal lesion.

GPR113/ADGRF3, GPR114/ADGRG5, and GPR128/ADGRG7: According to the Nephroseq database, *ADGRF3* expression is enriched in the glomerulus [35]. The Human Protein Atlas (HPA) database indicates that ADGRG5 is expressed at low levels in tubules [35] and *adgrg7b* is expressed based on RT-PCR predominantly in the adult kidney in zebrafish (~3-fold) [62]. In contrast, northern blot analysis of a variety of tissues revealed strong *Adgrf3* expression in the testis but none in the adult mouse kidney. This was confirmed by RT-PCR analysis, which detected additional expression only in circumvallate papillae [245]. CRISPR/Cas9-mediated *Adgrf3* KO mice exhibited no obvious phenotype, and associated in silico PCR (isPCR) analysis indicated that *Adgrf3* is not expressed in the mouse kidney but potentially in the human kidney [246]. A study analyzing *Adgrg7* KO mice reported that *Adgrg7* mRNA was highly and exclusively detected in intestinal tissues [247]. Therefore, we conclude that these aGPCRs most likely have no role in kidney development and physiology.

## 4. aGPCRs in Kidney Disease

AKI and CKD together account for a substantial and growing global health burden, with CKD affecting roughly 10–15% of adults in many populations and CKD-related mortality increasing over recent decades [19]. In the absence of therapies that can reliably restore lost nephron mass or reverse established renal dysfunction, there is growing interest in delineating molecular pathways that function both as mechanistic biomarkers and as tractable therapeutic targets to prevent or attenuate kidney injury. Emerging data place several aGPCRs within these pathways, often in cell type-specific and disease-context-dependent manners. Notably, several aGPCRs whose expression is known to be altered during kidney disease are known to be expressed by immune cells. For example, ADGRB1, ADGRE1, ADGRE3, ADGRE5, ADGRF5, ADGRG1, and ADGRG3 have been involved in the immune system [248] as well as the kidney (Table 1 and Table 2). According to snRNA-seq analysis, *Adgra3*, *Adgrc1*, *Adgrc2*, *Adgre1*, *Adgrf5*, *Adgrl1*, and *Adgrl4* are expressed in kidneys in immune cells [64]. Thus, it is important to determine which cell types in kidney disease show altered aGPCR expression, as it might reflect immune cell infiltration or activation of resident immune cells.

### 4.1. GPR56/ADGRG1 and GPR124/ADGRA2 in Diabetic Kidney Disease

**Figure 4:**
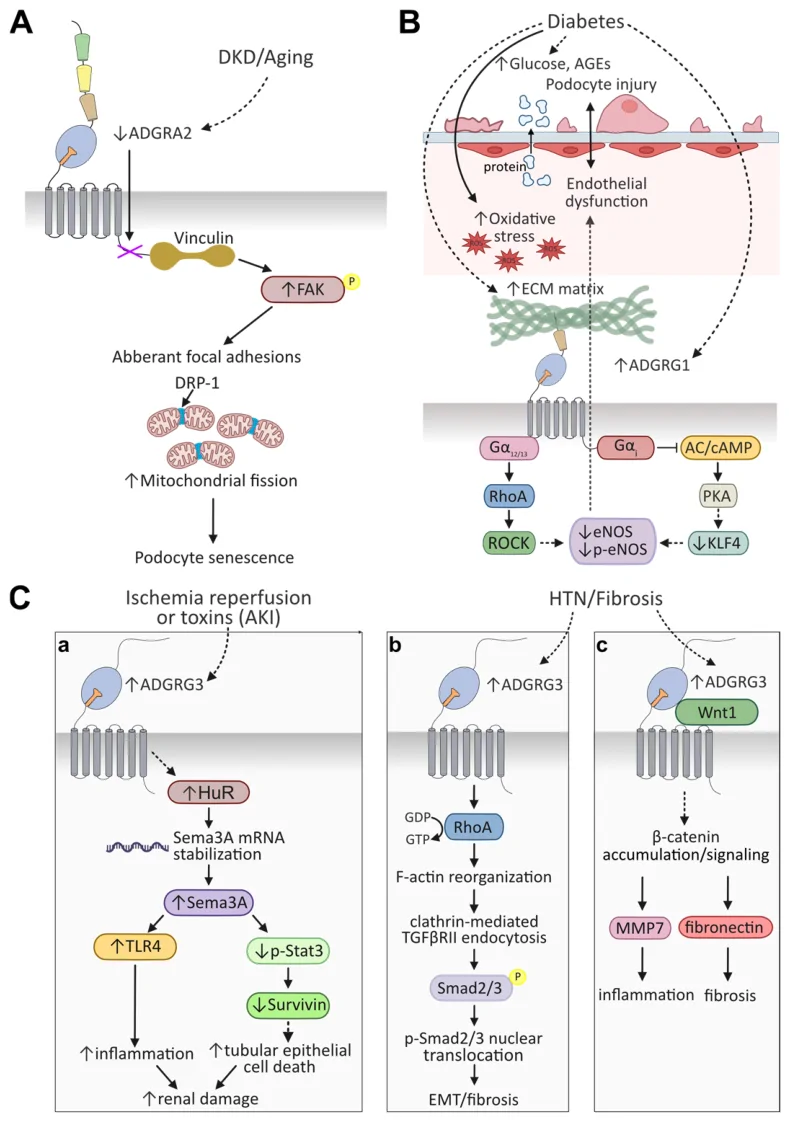
aGPCRs in kidney disease. (**A**) Role of GPR124/ADGRA2 in podocytes in diabetic kidney disease (DKD) and aging. In podocytes, ADGRA2 localizes to the plasma membrane and can directly bind to vinculin. In DKD and aging, ADGRA2 expression in podocytes is reduced. High-glucose treatment of podocytes decreases the levels of GPR124 and vinculin, leading to increased FAK phosphorylation and phosphorylation of the FAK target DRP1. This induces increased mitochondrial fission, exacerbating podocyte senescence. (**B**) Schematic illustration of GPR56/ADGRG1-mediated glomerular endothelial cell dysfunction in DKD. In diabetes, high glucose and advanced glycation end products (AGEs) promote ADGRG1 expression in glomerular endothelial cells (GECs). In GECs, ADGRG1 signals through Gα_12/13_ to activate the RhoA-ROCK pathway or Gα_i_ to inhibit the adenylyl cyclase–cAMP–PKA pathway, leading to reduced eNOS phosphorylation and expression, causing endothelial dysfunction and increased oxidative stress. This injury aggravates albuminuria and promotes podocyte damage, contributing to glomerular injury and DKD progression. (**C**) Overview of the roles of Gpr97/ADGRG3 in acute kidney injury (AKI), hypertensive nephropathy (HTN), and fibrosis. (**a**) In ischemia-reperfusion injury or toxin-induced AKI, ADGRG3 is upregulated and promotes HuR-dependent stabilization of semaphorin 3A (Sema3A) mRNA, leading to increased Sema3A expression. Elevated Sema3A then activates TLR4 signaling and downregulates STAT3 phosphorylation and survivin, driving inflammatory responses, tubular epithelial cell death, and worsening renal injury. (**b**) In hypertension-associated renal injury, ADGRG3 is upregulated and enhances RhoA-dependent F-actin reorganization, which facilitates clathrin-mediated endocytosis of TGFβRII and subsequent Smad2/3 phosphorylation and nuclear translocation, promoting epithelial–mesenchymal transition (EMT) and tubulointerstitial fibrosis. (**c**) ADGRG3 also regulates Wnt1/β-catenin signaling by binding Wnt1 to increase β-catenin accumulation and activity, leading to upregulation of MMP7 and fibronectin and thereby driving inflammatory responses and fibrotic remodeling.

Work in human biopsies and diabetic mouse models has revealed that several aGPCRs show reciprocal regulation in glomerular cells during diabetic kidney disease (DKD) [63,78]. GPR124/ADGRA2 has been studied more extensively in brain angiogenesis, but recent work points to a potential protective role in the glomerular compartment in DKD [63]. ADGRA2 protein is significantly reduced in glomeruli from DKD patients, where its expression localizes predominantly to podocytes; glomerular ADGRA2 levels correlate positively with the estimated glomerular filtration rate (eGFR) and negatively with serum creatinine, indicating that loss of ADGRA2 accompanies functional decline. Consistently, ADGRA2 protein is downregulated in podocytes of both type 1 (streptozotocin (STZ)-treated) and type 2 (db/db) diabetic mice, and high glucose or advanced glycation end products similarly suppress ADGRA2 in cultured human podocytes. Podocyte-specific deletion of *Adgra2* in these diabetic models aggravates kidney injury, with increased albuminuria, mesangial expansion, podocyte damage, reduced endothelial fenestrations, and enhanced podocyte senescence. In line with this, senescence markers such as SA-β-gal, p53, p21, and p16 were upregulated in podocytes. This suggests that ADGRA2 may restrain podocyte senescence in the diabetic milieu. Mechanistic experiments using genetic and biochemical approaches show that ADGRA2 binds vinculin and restrains focal adhesion kinase (FAK) activation; FAK signaling in turn influences mitochondrial dynamics via dynamin-related protein 1 (DRP1), providing a plausible route by which ADGRA2 protects podocytes from senescence under diabetic stress (Figure 4A).

*GPR56*/*ADGRG1*, expressed in DTs and PTs [249] as well as in glomeruli, is increased in glomeruli of patients with DKD [78]. In line with the human data, *Adgrg1* expression is upregulated in an advanced type 1 diabetic nephropathy model (STZ-treated eNOS-deficient mice) in GECs [78] and in vitro exposure of GECs to high glucose raises ADGRG1 protein abundance [250]. Although *Adgrg1* KO mice did not display any gross renal defects, upon induction of diabetes in *Adgrg1* KO mice with STZ, kidney injury signatures were reduced. This included reduced albuminuria, mesangial matrix accumulation, glomerular hypertrophy, and podocyte foot process effacement. Using biochemical and cellular techniques, *Adgrg1* loss was found to restore the expression of the transcription factor “Krüppel-like factor 4” (KLF4) and “endothelial nitric oxide synthase” (eNOS) in GECs of diabetic mice, thereby reducing oxidative stress known to play a key role in DKD pathogenesis (Figure 4B). Mechanistically, it has been proposed that the activation of ADGRG1 by brief stimulation with the ECM molecule collagen III activates the Gα_12/13_-RhoA-ROCK pathway, resulting in reduced eNOS phosphorylation. Furthermore, activation of Gα_i_ via ADGRG1 might control the total expression of eNOS over time by inhibiting cAMP/protein kinase A (PKA) signaling to regulate the expression of KLF4. These data indicate that ADGRG1 functions as a maladaptive GEC receptor whose upregulation promotes oxidative damage and proteinuria, such that its genetic ablation improves diabetic glomerular outcomes [78].

### 4.2. GPR97/ADGRG3 in Acute Kidney Injury

GPR97/ADGRG3 has been implicated as a pro-injury mediator in multiple forms of AKI (Figure 4C). It is expressed in several renal parenchymal cell types in mouse and human kidneys [79]. Importantly, ADGRG3 protein is markedly increased in kidney biopsies from patients with biopsy-proven acute tubular necrosis, the most common intrinsic cause of AKI [79]. Similarly, in mouse renal ischemia-reperfusion injury (IRI), an AKI model, *Adgrg3* mRNA and protein are strongly upregulated, especially in PTs and DTs 24 h post-injury [79]. Upon IRI induction in *Adgrg3* KO mice, lower serum creatinine and blood urea nitrogen levels were observed, in addition to less tubular damage on histology, reduced tubular cell death, and inflammatory infiltrates compared with wild-type mice.

Similar beneficial effects of Adgrg3 deficiency have been reported in additional AKI settings, including a cisplatin-mediated in vivo AKI model as well as in vitro oxygen-glucose deprivation (OGD) and CoCl-based hypoxia induction, primary hallmarks of AKI. Mechanistically, ADGRG3 has been shown to promote human antigen R (HuR)-dependent stabilization of semaphorin-3A (Sema3a) mRNA under hypoxic conditions, leading to increased SEMA3A expression [79]. SEMA3A in turn drives Toll-like receptor 4 (TLR4)-mediated inflammatory signaling and tubular cell death and negatively regulates “signal transducer and activator of transcription 3” (STAT3)–survivin pathway that is protective in AKI, providing a plausible explanation for the milder injury phenotype in *Adgrg3* KO mice. These findings position ADGRG3 as a pro-injury factor in AKI, but the extent to which this pathway contributes to human disease remains to be clarified.

### 4.3. GPR97/ADGRG3 in Hypertensive Nephropathy and Fibrosis

Beyond AKI, ADGRG3 also contributes to hypertensive kidney injury and fibrogenesis. In patients with hypertensive nephropathy and in deoxycorticosterone acetate (DOCA)–salt hypertensive mice, ADGRG3 expression is increased, alongside activation of β-catenin signaling. In human disease, ADGRG3 levels correlate positively with serum creatinine and proteinuria and negatively with the eGFR [251]. In hypertensive mice, ADGRG3 upregulation is particularly evident in PT epithelial cells, and genetic deletion of *Adgrg3* ameliorates mesangial matrix expansion and tubulointerstitial fibrosis [252]. In vitro, silencing *Adgrg3* in PT cells reduces transforming growth factor (TGF)-β1–induced inflammation and fibrotic marker expression. Mechanistic studies indicate that ADGRG3 promotes TGF-β1 receptor endocytosis via RhoA, leading to Smad2 activation and fibrosis [251]. In addition, Wnt1 can directly bind ADGRG3 in a TGF-β1-dependent manner to enhance β-catenin and matrix metalloproteinase (MMP) 7 expression, further driving fibrogenesis [252]. Crosstalk between RhoA and β-catenin—through effects on the actin cytoskeleton or direct binding of phosphorylated RhoA to β-catenin—likely integrates these pathways, although the precise downstream network remains to be resolved.

## 5. Discussion

The kidney performs a wide range of essential physiological functions, including filtration, reabsorption, secretion, and the maintenance of fluid and electrolyte homeostasis. These functions are highly compartmentalized among structurally and molecularly distinct segments of the nephron, each defined by a characteristic repertoire of receptors, ion channels, and transport proteins [253,254,255,256,257,258,259,260,261,262]. In addition, nephron epithelial cells exhibit pronounced apical-basolateral polarity [263,264], and many tubular cells extend a single primary cilium into the lumen, where it serves as a mechano- and chemosensory organelle [13,14,265,266,267]. So far, the expression of 25 of 33 aGPCRs has been detected in the kidney during development, physiology, and disease (Table 1 and Table 2). Given their large and structurally diverse extracellular domains [44], aGPCRs are well suited to detect a broad range of extracellular cues and transduce these into distinct cellular behaviors. Consistent with this concept, ADGRF5 has been reported to localize to the apical membrane of IC-As in the CD, where it contributes to acid–base regulation by modulating the apical trafficking of V-ATPases [33]. In addition, aGPCRs might be interacting with other membrane proteins, modulating their function. Notably, the atypical, 11-transmembrane receptor polycystin-1 with several aGPCR-like features (GAIN-like domain, *Stachel*, GPS, autoproteolytic cleavage into NTF and CTF, heterotrimeric G protein signaling) and the calcium-permeable, non-selective cation channel polycystin-2 physically associate to form a membrane-localized polycystin complex that integrates extracellular or mechanical cues with intracellular signaling, which is relevant to renal tubular architecture and the pathogenesis of autosomal dominant polycystic kidney disease [268,269].

Over the past decade, the study of aGPCRs in the kidney has progressed from largely descriptive associations based on RNA-expression profiles to functional and mechanistic evidence. These studies suggest that selected aGPCRs participate in kidney development and homeostasis, while genetic or pharmacological perturbation of their expression or signaling can influence renal function and disease progression. The spatially restricted expression pattern of individual aGPCRs within specific renal cell types or nephron segments allows prediction of which distinct, context-dependent physiological and pathological processes are regulated. For example, ADGRC1 is expressed very early in ureteric and tubular epithelial cells and has been implicated in polarity during nephrogenesis and cystogenesis [85] while ADGRF5 is expressed in podocytes and IC-As and is required for the maintenance of glomerular endothelial integrity [76] and control of acid secretion in the CD [33], respectively. However, so far, only 7 of the 25 renally expressed aGPCRs have had their renal function identified.

There might be a simple reason why we have so far elucidated the renal function of only a few aGPCRs using KO mouse models. Besides the classical challenges (e.g., genetic compensation (e.g., *Pax2*/*Pax8* [270]), functional redundancy, developmental adaptation, incomplete gene disruption (*adgrg6*, myelination vs. cardiac phenotype [46,271]; *Adgrf5*, no overt renal phenotype vs. impaired renal function [33,74]), background and environmental effects), there are several kidney-specific challenges. Notably, after loss of one kidney or reduction in nephron mass, the remaining kidney can undergo compensatory growth and increase single-nephron filtration. In general, the functional reserve of the kidney is significant, and thus phenotypes can often only be observed during salt or water loading, acid–base stress, diuretic treatment, high-protein feeding, ischemia, hypertension, or nephrotoxic injury. While *Adgrg1* and *Adgrg3* KO mice did not exhibit an obvious renal phenotype, these mice were protected from renal damage upon AKI and during DKD [78,79]. Importantly, segment-specific impairment can also be compensated by another nephron segment by altered delivery, reabsorption, or secretion in downstream segments. In PTs, individual deletion of NHE3 (Sodium-Hydrogen Exchanger 3) and aquaporin-1, both required for sodium and water reabsorption, resulted in reduced proximal fluid reabsorption, but Na^+^ loss was minimal, largely because GFR was secondarily reduced via tubuloglomerular feedback, which normalizes distal delivery. In some settings (e.g., Nhe3^+/−^ mice and hypothyroid rats), downstream segments also increase their reabsorptive activity (e.g., upregulation of NKCC2 and ENaC), further preserving Na^+^ balance [272,273,274,275]. Moreover, the generated phenotype might be so specific that conventional readouts such as gross morphology, GFR, serum creatinine, blood urea nitrogen, urine output, and electrolyte concentrations might not detect it. For example, constitutive KO of *Adgrf5* in mice was initially reported to have no phenotype [74]. However, upon closer inspection, enhanced urinary acidification with increased blood pH was observed [33].

In addition to renal-specific considerations, there are also some aGPCR-specific considerations: (i) Maternal compensation. For almost all aGPCRs in zebrafish, it has been reported that maternal mRNAs are loaded into the oocyte before fertilization [62], potentially compensating for zygotic gene loss and preventing early developmental phenotypes [181]. (ii) Non-cell autonomous function. While RNA expression data can be utilized to predict function to perform more specialized investigations, it is important to note that the non-covalent association of the NTF to the CTF on the cell surface makes it possible for the NTF to act in a non-cell autonomous manner [46]. Thus, the NTF of a highly specifically expressed aGPCR might have an unexpected function such as controlling the function of a downstream nephron segment. ADGRG6 is, for example, expressed specifically in endocardial cells in the heart but can bind to cardiomyocytes [46] and controls cell–cell adhesion of cardiomyocytes [181]. (iii) Lack of antibodies. There is a significant lack of validated antibodies against aGPCRs. Current expression information of aGPCRs is largely based on RNA-based approaches—RNA-seq, microarray, in situ hybridization, and quantitative RT-PCR (Table 1). Thus, the localization of shed NTFs cannot be determined. Furthermore, the subcellular localization of aGPCRs cannot be determined. This would be of particular interest as renal epithelial cells are highly polarized and organized into discrete functional domains. The subcellular distribution of a receptor—apical, basolateral, ciliary, intracellular, or matrix-associated—largely dictates which signals it can perceive and how it shapes cellular behavior. For ADGRA3 and ADGRF5, it is known that they are expressed in basolateral and apical membranes, respectively. Notably, these localizations are in alignment with their identified functions, i.e., polarized cell division during cystogenesis [65] and acid–base balance of urine [33] on the luminal side.

The identification of aGPCRs as a novel class of diagnostic and therapeutic targets could have substantial clinical and socioeconomic implications given the increasing global burden of kidney disease. The incidence of kidney disease is rising worldwide [276,277], with AKI occurring in approximately one-third of children and one-fifth of adults during hospitalization [277]. CKD is now among the ten leading causes of death globally and accounts for approximately one death every 20 s [276,278]. Moreover, the kidney’s substantial functional reserve and the compensatory hyperfiltration of surviving nephrons can preserve global renal function until substantial structural damage has occurred, thereby limiting the sensitivity of conventional markers for early disease detection [279,280]. These considerations underscore the need to identify novel biomarkers and therapeutic strategies that enable earlier diagnosis and more effective intervention. An expanding body of evidence indicates that aGPCR expression is dysregulated during the transition from renal homeostasis to disease (Table 2), supporting their potential utility as biomarkers and drug targets. However, there are so far only two aGPCRs, ADGRG1 and ADGRG3, for which functional studies in mouse models have demonstrated a causal contribution to kidney disease.

Four features make aGPCRs particularly attractive therapeutic targets in kidney disease. First, their membership in the GPCR superfamily, which already comprises molecular targets for more than one-third of currently marketed therapeutic drugs [281]. Second, the recent expansion of available aGPCR structural information is beginning to reveal receptor-specific ligand-binding sites, activation mechanisms, and opportunities for rational drug design [282]. Third, several aGPCRs have progressed from being merely associated with renal disease on the basis of differential expression to being mechanistically implicated in defined pathological processes, thereby establishing them as concrete therapeutic candidates. After demonstrating that *Adgrg1* KO mice exhibited a significant reduction in diabetes-induced albuminuria and glomerular injury and *Adgrg3* KO mice had significantly less renal injury in AKI models, it would be of great interest to validate the results with cell-type-specific and inducible KO models and to utilize selective agonists and antagonists as a therapeutic approach. Importantly, there is significant progress in identifying small molecules capable of modulating ADGRG1 signaling (selective antagonist, dihydromunduletone; partial agonist, 3α-DOG) [282,283,284]. Fourth, there are several ways to activate aGPCRs. *Stachel*-derived peptidomimetics can be used to identify agonists. The large extracellular domain can be targeted by antibodies, nanobodies, and monobodies. These approaches have been used to modulate ADGRG1, ADGRE2, ADGRE5, and ADGRL3 signaling [70,281,285,286,287,288]. Moreover, PrPC has been identified as an endogenous agonistic ligand of ADGRG6, which can activate ADGRG6-dependent signaling associated with peripheral myelin maintenance [156].

## 6. Conclusions

We conclude that defining the functional roles of aGPCRs in renal development, homeostasis, and disease represents a promising and rapidly emerging area of investigation. A deeper understanding of their cell type- and segment-specific expression, ligand interactions, activation mechanisms, and downstream signaling pathways may reveal previously unrecognized determinants of kidney function and pathology. These insights could allow the utilization of the immense untapped therapeutic potential of aGPCRs, establishing them as a therapeutically tractable receptor class and supporting the development of selective agonists, antagonists, antibodies, or ligand-mimetic strategies for the treatment of kidney disease. For this purpose, it is necessary to identify the subcellular localization of NTF and CTF of aGPCRs by validated antibodies or by generating knock-in reporter cell or mouse lines to track endogenous aGPCR protein. Note that while some anti-aGPCR antibody-based stainings appear to show highly specific staining patterns, validation is strictly necessary. For multiple proteins, including angiotensin II receptor and cannabinoid receptor, highly specific-appearing staining patterns were shown to be non-specific by utilizing KO animals [289,290]. In the future, it will be important to predict potential renal functions of aGPCRs and design accordingly detailed analyses of renal morphology and function, and to challenge kidney function by overcoming the functional reserve of the kidney and compensatory mechanisms such as tubuloglomerular feedback control to reveal the function of aGPCRs.

## Figures and Tables

**Table 3:** Ligands and Interaction Partners of Renal aGPCRs.

aGPCR	Ligands and Interaction Partners	Gα Coupling	Signaling in the Kidney	Refs.
ADGRA1	Orphan; no ligand or partner identified. The only aGPCR lacking a GAIN domain, and therefore not activated by a tethered agonist	—	—	[44]
ADGRA2	RECK; vinculin; ITSN1; ELMO; DOCK; FZD; LRP5; LRP6; DLG1; α_V_β_3_-integrin; glycosaminoglycans	—	Vinculin binding → inhibition of FAK signaling → protection from senescence and mitochondrial injury via DRP1 and microtubules	[63,91,92,93,94,95]
ADGRA3	Orphan (tethered-agonist activation after GPS autoproteolysis); *Stachel* peptide; hesperetin; DLG1 (kidney epithelium, MDCK); Dvl (planar polarity)	Gα_i_, Gα_s_	DLG1-dependent basolateral trafficking → apicobasal polarity and oriented cell division (cyst suppression)	[65,89,96,97]
ADGRB1	phosphatidylserine (via TSRs; apoptotic-cell engulfment); LPS of Gram-negative bacteria; RTN4-receptor; CD36; Par3/Tiam1; alpha(v)beta (5); variety of PDZ domains from synaptic proteins; BAIAP2; α_v_β_5_-integrin. ELMO	Gα_12/13_	Vasculostatin (cleaved BAI1 ectodomain) is anti-angiogenic → reduced VEGF and tumor microvessel density in renal cell carcinoma; (ELMO/DOCK180–Rac1 drives efferocytosis)	[98,99,100,101,102,103,104,105]
ADGRB2	Largely orphan; tethered agonist with high basal activity; TIP-1		—	[106]
ADGRB3	C1QL1–C1QL4 (complement C1q-like proteins); stabilin-2; ELMO (myoblast fusion); C1ql3; RTN4-receptor	—	—	[100,107,108,109,110,111]
ADGRC1	Homophilic CELSR1–CELSR1 adhesion via cadherin repeats. Partners: VANGL2 (kidney development)	—	Planar cell polarity (VANGL2/Frizzled/Dvl) → oriented cell division, ureteric-tree branching and glomerular maturation	[67,85,112]
ADGRC2	GARS1; homophilic interactions	—	—	[113,114]
ADGRC3	Oligomeric β-amyloid; homophilic interactions; dystroglycan	—	Planar cell polarity → urinary-tract morphogenesis (zebrafish)	[114,115]
ADGRE5	CD55; α_5_β_1_-integrin; chondroitin sulfate; β-catenin; DLG1; *Stachel* peptide	Gα_13_, Gα_q_, Gα_s_	—	[40,116,117,118,119,120,121,122,123,124,125]
ADGRF1	Occludin; laminin-111; synaptamide	Gα_q_, Gα_s_, Gα_i_, Gα_12_, Gα_13_	—	[126,127,128,129,130]
ADGRF5	FNDC4 (soluble); *Stachel* peptide, Surfactant Protein-D	Gα_q_, Gα_11_	Gα_q_ → apical V-ATPase trafficking in type A intercalated cells → inhibition of renal acid secretion	[33,75,131,132,133,134]
ADGRG1	COL3A1; TG2; heparin; phosphatidylserine; tetraspanins CD9/CD81; 17α-hydroxypregnenolone; progastrin	Gα_12/13_, Gα_q/11_	In glomerular endothelium, ADGRG1 suppresses eNOS via PKA/KLF4 → promotes diabetic kidney disease; (Gα_12/13_ → RhoA)	[38,78,135,136,137,138,139,140,141,142,143,144,145,146] *
ADGRG3	Glucocorticoids (beclomethasone, cortisol; exogenous agonists); 3-acetoxydihydrodeoxygedunin (3-α-DOG); tethered agonist; CD177-associated membrane proteinase 3 (mPR3); astragaloside IV; L-Phe	Gα_o_, Gα_13_, Gαs, Gα_i_	GPR97 → Sema3A signaling (HuR-stabilized) → exacerbation of AKI	[37,147,148,149,150,151]
ADGRG4	Orphan	—	—	—
ADGRG6	Collagen IV; laminin-211; PRNP; tethered agonist; progesterone; 17-hydroxyprogesterone	Gα_s_, Gα_q_, Gα_12_, Gα_i_	—	[47,48,152,153,154,155,156,157]
ADGRL2	FLRT2; TMC1; LRG1; HSP90; caveolin-1	Gα_q/11_, Gα_12/13_, Gα_s_	—	[158,159,160,161,162,163,164,165]
ADGRL3	FLRT3; α-latrotoxin; tethered agonist	Gα_12_, Gα_13_, Gα_s_, Gα_i_, Gα_q/11_	—	[166,167,168,169,170,171]
ADGRL4	KU80; SPTB; tethered agonist	Gα_q_	With ADGRF5/GPR116, maintenance of glomerular endothelial fenestration (double KO → renal thrombotic microangiopathy); [Notch/DLL4 in tumor angiogenesis]	[74,172,173]
ADGRV1	Usherin; EMILIN3; whirlin; PDZD7; CIB2; tethered agonist	Gα_s_, Gα_q/11_,	—	[174,175,176,177,178,179,180]

Abbreviations: 3-α-DOG, 3-acetoxydihydrodeoxygedunin; Aβ, Amyloid-β; AKI, Acute kidney injury; BAIAP2, BAI1-associated protein 2; C1QL1–C1QL4/C1ql3, Complement C1q-like proteins 1–4; CD36/CD55/CD9/CD81/CD177, Clusters of differentiation 36/55/9/81/177; CIB2, Calcium and integrin-binding family member 2; COL3A1, Collagen type III α1 chain; DLL4, Delta-like ligand 4; DLG1, Discs large homolog 1; DOCK/DOCK180, Dedicator of cytokinesis; DRP1, Dynamin-related protein 1; Dvl, dishevelled; ELMO, Engulfment and cell motility protein; ELMILIN3, Elastin microfibril interfacer 3; eNOS, Endothelial nitric oxide synthase; FAK, Focal adhesion kinase; FLRT2/FLRT3, Fibronectin leucine-rich transmembrane protein 2/3; FNDC4, Fibronectin type III domain-containing protein 4; FZD, Frizzled; GAIN, GPCR autoproteolysis-inducing domain; GARS1, Glycyl-tRNA synthetase 1; HSP90, Heat-shock protein 90; HuR, Human antigen R (ELAVL1); ITSN1, Intersectin 1; KLF4, Krüppel-like factor 4; L-Phe, L-phenylalanine; LPS, Lipopolysaccharide; LRG1, Leucine-rich α2-glycoprotein 1; LRP5/LRP6, Low-density lipoprotein receptor-related protein 5/6; mPR3, Membrane-bound proteinase 3; Par3, Partitioning-defective 3; PDZD7, PDZ domain-containing protein 7; PKA, Protein kinase A; PRNP, Prion protein gene; Rac1, Ras-related C3 botulinum toxin substrate 1; RCC, Renal cell carcinoma; RECK, Reversion-inducing cysteine-rich protein with Kazal motifs; RhoA, Ras homolog family member A; RTN4, Reticulon 4; Sema3A, Semaphorin 3A; SP-D, Surfactant protein D; SPTB, Spectrin β, erythrocytic; TG2, Transglutaminase 2; Tiam1, T-lymphoma invasion and metastasis-inducing protein 1; TIP-1, Tax-interacting protein 1; TMC1, Transmembrane channel-like protein 1; TSR, Thrombospondin type 1 repeat; V-ATPase, Vacuolar-type H^+^-ATPase; VANGL2, Van Gogh-like planar cell polarity protein 2; VEGF, Vascular endothelial growth factor. Refs.: References. * Pre-print, not peer-reviewed.

## Data Availability

No new data were created or analyzed in this study. Data sharing is not applicable to this article.

## Abbreviations

The following abbreviations are used in this manuscript:

7TM	7 transmembrane
aGPCR	Adhesion G protein-coupled receptor
AKI	Acute kidney injury
cAMP	3′,5′-cyclic adenosine monophosphate
CD	Collecting duct
CFTR	Cystic Fibrosis Transmembrane Conductance Regulator
CKD	Chronic kidney disease
CME	Clathrin-mediated endocytosis
CNS	Central nervous system
CNT	Connecting tubule
CTF	C-terminal fragment
DCT	Distal convoluted tubule
DKD	Diabetic kidney disease
DLG1	Discs large homolog 1
DT	Distal tubule
E	Embryonic day
eGFR	Estimated glomerular filtration rate
ENaC	Epithelial sodium channel
eNOS	Endothelial nitric oxide synthase
FAK	Focal adhesion kinase
FGF	Fibroblast growth factor
GAIN	GPCR autoproteolysis-inducing
GBM	Glomerular basement membrane
GDNF	Glial cell line-derived neurotrophic factor
GEC	Glomerular endothelial cell
GFB	Glomerular filtration barrier
GPCR	G protein-coupled receptor
GPS	GPCR proteolysis site
IC	Intercalated cell
IC-A	Intercalated cell, type A
KIM-1	kidney injury molecule-1
KLF4	Krüppel-like factor 4
KO	Knockout
LGN	Leucine–glycine–asparagine
LOH	Loop of Henle
MDCK	Madin–Darby canine kidney cell
MO	Morpholino
NTF	N-terminal fragment
NuMA	Nuclear and mitotic apparatus
PC	Principal cell
PCP	Planar cell polarity
PCR	Polymerase chain reaction
PEC	Parietal epithelial cell
PT	Proximal tubule
RhoA	Ras homolog family member A
RT-PCR	Real-time PCR
STZ	Streptozotocin
TGF	Transforming growth factor
TIM-1	T cell IgG and mucin containing 1
UB	Ureteric bud
V-ATPase	Vacuolar-type H^+^-ATPase
Wnt	Wingless-related integration site
